# Improving Electrical Conductivity of Commercially Pure Aluminium: The Synergistic Effect of AlB8 Master Alloy and Heat Treatment

**DOI:** 10.3390/ma18020364

**Published:** 2025-01-15

**Authors:** Yusuf Zeybek, Cemile Kayış, Ege Anıl Diler

**Affiliations:** 1 Graduate School of Natural and Applied Sciences, Ege University, Izmir 35040, Turkey; 91230000163@ogrenci.ege.edu.tr; 2 Volt WEG Group, Izmir 35735, Turkey; 3 Department of Mechanical Engineering, Ege University, Izmir 35040, Turkey

**Keywords:** commercially pure aluminium, electrical conductivity, aluminium–boron master alloy, grain-coarsening heat treatment, efficiency of electrical motor

## Abstract

This study aims to enhance the electrical conductivity of commercially pure aluminium by minimizing impurities and grain boundaries in its microstructure, ultimately improving the efficiency of electric motors constructed from rotors with squirrel cages made from this material. For this purpose, an aluminium–boron (AlB8) master alloy was added to aluminium with a purity of 99.7%, followed by the application of a grain-coarsening heat treatment to the rotors. To obtain commercially pure aluminium with boron additions of 0.05% and 0.1% by weight, specific amounts of the AlB8 master alloy were added into aluminium with a purity of 99.7%. Using these materials, squirrel cage components of rotors were produced via the high-pressure die-casting method. Subsequently, a grain-coarsening heat treatment of the rotors was performed at temperatures of 450 °C, 500 °C, and 550 °C, with holding times of 2, 6, and 10 h. The Box–Behnken design, which is based on statistical experimental design and response surface methodology, was employed to investigate the effects of adding boron and varying the heat treatment temperature and holding time on the electrical conductivity of commercially pure aluminium. The results showed that the synergistic effect of adding boron at 0.05 wt.% and applying the grain-coarsening heat treatment at a temperature of 550 °C for a holding time of 10 h significantly enhanced the electrical conductivity of commercially pure aluminium, increasing it from 60.62% IACS to 63.1% IACS. Correspondingly, the efficiency of the electric motor increased from 90.35% to 91.53%. These findings suggest that this hybrid method not only enhances the electrical conductivity of commercially pure aluminium but also has strong potential to improve its other properties, such as thermal conductivity. This will lead to products composed of components manufactured from the materials exhibiting better performance characteristics, such as increased efficiency and extended service life. Consequently, this innovative method will contribute economically and environmentally by facilitating the manufacture of high-performance products.

## 1. Introduction

Energy efficiency has become an increasingly important issue because of the rising energy demands and the negative impact of greenhouse gases emitted directly from the burning of fossil fuels on climate change. Over 50% of the global electricity consumed is used by electric motors [1] due to their extensive utilization in industrial and commercial applications such as electric vehicles, aircraft carriers, robots, and machine tools [2,3,4,5]. However, electric motors are a more sustainable choice due to their higher energy efficiency [6], reduction in greenhouse gas emissions [7], and less maintenance [8] compared to internal combustion engines. This is why it is crucial to prioritize improving energy efficiency in electric motor equipment and technologies as a key strategic foresight.

Electric motors come in various types, each designed for specific applications and with their own unique characteristics. Alternating current (AC) motors and direct current (DC) motors are the two main types of motors used in industrial applications. AC motors consist of induction (asynchronous) motors and synchronous motors, while DC motors include brushed and brushless varieties. These motors are chosen for their distinct advantages based on various factors such as performance requirements (power, torque, speed, etc.) [9], energy efficiency [10], and cost-effectiveness [11]. Brushed DC motors are known for their simple design and cost-effectiveness, while brushless DC motors offer higher efficiency and performance than brushed DC motors [12]. On the other hand, there are drawbacks to DC motors such as a limited range of speed operation, insufficient overload capacity, the need for frequent maintenance, and power loss in the field circuit [13]. Synchronous motors, particularly permanent magnet synchronous motors, are preferred over DC motors due to their high power density, high speed, small size, and high reliability [14]. In addition, synchronous motors offer a higher efficiency and power factor than induction (asynchronous) motors [13]. However, induction motors are the most commonly used type of motor in industrial applications, including electric vehicles, due to their advantages of high efficiency, low cost, reliability, robustness, and low maintenance requirements [15,16,17,18,19]. Although induction motors have many advantages and are among the high-efficiency electric motors, they can still experience certain losses, including stator, rotor, iron core, mechanical, and stray losses. Overall, 15–25% of motor losses occur in the rotor, which is one of the most crucial components of induction motors [20]. There are two types of induction motors based on rotor structure: squirrel cage and slip ring (wound) induction motors. Over 90% of induction motors are of the squirrel cage rotor type, primarily due to their simple and robust structure [21]. A squirrel cage rotor consists of a laminated iron core, conductor bars, and end rings. The number of rotor slots and bar geometry have a strong impact on the performance of squirrel cage induction motors [22,23,24,25,26]. Another significant factor influencing the efficiency of squirrel cage induction motors is the conductivity of the bar material. Konda et al. [27] conducted a study comparing the efficiency of squirrel cage induction motors with copper rotors to those with aluminium rotors. The results showed that the efficiency of motors with copper rotors was 87%, demonstrating an 11% improvement over motors with aluminium rotors. Özsoy et al. [28] studied the effect of rotor slot geometry and squirrel cage material on motor performance in induction motors by conducting analyses with the finite element method. They compared aluminium and copper cage motors with six different rotor slot geometries and found that by using copper bars with a certain rotor slot geometry, they achieved an efficiency of 92.12%. These studies indicated that squirrel cage induction motors with copper rotors were more efficient than those with aluminium rotors. However, some studies have suggested that although the efficiency of copper rotors is effective on the efficiency of aluminium rotors, copper rotors may cause some drawbacks. Marfoli et al. [29] investigated the impact of various rotor slot geometries of copper and aluminium bars on the efficiency of squirrel cage induction motors. They suggested that the direct replacement of aluminium with copper resulted in higher efficiency, although it also increased the starting current. Liu et al. [30] conducted a detailed analysis of different materials used for rotor bars in squirrel cage induction machines. They reported that the silver rotor provided the highest efficiency under all tested loads and machines, with an efficiency enhancement of 0.20% to 1.55% in comparison to an aluminium rotor. Additionally, they stated that both aluminium and silver have lower melting points than copper, which results in less thermal stress on casting dies and makes rotor bar manufacturing easier. However, they noted that the high cost of silver can limit its practical usage in many applications currently. Ocak [31] examined the influence of using copper and aluminium bar materials and the impact of the number of rotor bars and altering rotor slot geometry on the performance of squirrel cage induction motors. The copper cage demonstrated higher efficiency compared to the aluminium cage; however, it was noted that the cost of copper cage injection is prohibitive, leading to the continued popularity of aluminium alternatives in both design and production. Although relevant studies in the literature suggest that copper squirrel cage rotors are more efficient and have higher electrical conductivity than aluminium ones, they have some drawbacks depending on the copper material itself and the die-casting process in the production of squirrel cage rotors. Processing copper presents greater challenges and costs due to its higher melting point, thermal conductivity, density, and cost per unit [32]. Compared to an aluminium rotor, manufacturing a copper rotor requires the process to be carried out at a higher temperature due to its high melting point. This results in electrical steel laminates in the rotor experiencing more significant and faster thermal shock, leading to a greater reduction in the efficiency of the rotor [33,34,35]. Thermal shock frequently causes electrical steel laminates to short circuit with bar conductors in contact areas. This shorting results in a deterioration of inter-bar resistance, ultimately causing the apparition of inter-bar currents, particularly noticeable in induction motors manufactured from die-cast copper [33,36]. During the die-casting process of copper rotors, issues such as copper contamination and the formation of cuprous oxide may also arise. Exposure of molten copper to environmental oxygen results in the formation of cuprous oxide, which subsequently decreases the conductivity of the rotor bars [37]. Furthermore, due to its high melting point, copper is prone to contamination by impurity elements from the die material and steel laminations it contacts. This contamination leads to a decrease in the electrical conductivity of the rotor, ultimately reducing the efficiency of the electric motor [38,39]. Due to the lower melting point of aluminium compared to copper, these drawbacks are less likely to be encountered in the manufacturing of aluminium rotors. Aluminium also offers advantages such as being low-cost and lightweight. Furthermore, global aluminium reserves are greater than copper, and aluminium has a higher recycling rate [40]. As a result of all these factors, aluminium remains a popular alternative for manufacturing electric motor rotors, making the enhancement of its electrical conductivity a crucial strategy.

It is common knowledge that pure aluminium exhibits superior electrical conductivity compared to its alloys. The conductivity of pure aluminium and aluminium alloys can be improved by various methods such as reducing impurities by adding master alloys and rare earth elements. Studies investigating the effect of rare earth elements, such as cerium (Ce), lanthanum (La), and yttrium (Y), on electrical conductivity have shown that the electrical conductivity of aluminium can be increased due to the ability of these elements to remove impurities from aluminium [41,42]. On the other hand, relevant studies have indicated that high amounts of Ce and La can lead to decreased electrical conductivity in commercially pure aluminium (CP-Al) [42,43,44]. While these studies have indicated that adding rare earth elements can improve the electrical conductivity of CP-Al, it is crucial to note that using rare earth elements is a costly process. A cost-effective alternative for enhancing the electrical conductivity of CP-Al is to use master alloys, such as aluminium–boron (Al–B), instead of expensive rare earth elements. Trace elements (impurities) and their amounts in the microstructure are one of the main factors that can significantly impact the electrical conductivity of aluminium. Even though CP-Al contains a restricted amount of dissolved trace elements, these elements can impact electrical conductivity. This can be attributed to the following factors [45]. One reason is that the tendency of these elements to form solid solutions with aluminium can lead to lattice distortion. This distortion can impede the flow of electrons, ultimately affecting the conductivity of the material. Another factor is that trace elements exhibit high activity in molten aluminium, easily reacting with aluminium to form compounds between trace elements and aluminium. This reaction reduces the number of free electrons during electronic transmission. The addition of boron induces a transition of trace elements from a solid solution to the formation of boride compounds. These compounds then settle at the bottom of molten aluminium, which effectively minimizes their negative impact on the electrical conductivity of aluminium [46]. Al–B master alloys can be highly effective in enhancing the electrical conductivity of CP-Al through the aforementioned mechanism. For this purpose, some studies have focused on the influence of Al–B master alloys on improving the electrical conductivity of CP-Al. In these studies, Al–B master alloys containing varying amounts of boron, and consequently including aluminium diboride (AlB_2_) and aluminium dodecaboride (AlB_12_) compounds, were utilized during the casting process of CP-Al. Karabay and Uzman [47] studied the impact of inoculating trace elements by adding master alloys containing 3% AlB_2_, 4% AlB_12_, and 5% AlB_12_ on the electrical conductivity of 99.6% aluminium. The incorporation of 3% AlB_2_ was reported to enhance the electrical conductivity of aluminium by mitigating the detrimental effects of trace elements. Xu et al. [48] investigated the influence of Al–3B master alloy on the electrical conductivity of 1070 aluminium (99.7%). The addition of Al–3B master alloy had little impact in improving the electrical conductivity of 1070 aluminium. However, Cui et al. [45] suggested that the electrical conductivity of AA1070 aluminium significantly improved by adding Al–6B master alloys. This enhancement was attributed to a reduction in the concentration of trace metal elements in the molten aluminium, resulting from the formation of metal borides that settled at the bottom of the melt. In addition to this benefit, Al–B master alloys are also utilized as grain refiners to decrease the grain size of CP-Al, such as in Al 99.7% [45,48,49,50]. However, it is well recognized that there is a substantial relationship between grain size and electrical conductivity, with a decrease in grain size potentially impacting the electrical conductivity of metals negatively [51,52]. As the amount of master alloy added to aluminium increases, the grain size decreases [53,54,55]. This can negatively impact the positive effect of master alloy in improving the electrical conductivity of aluminium by reducing impurities. It should be noted that various master alloys, such as Al–B, Al–Ti–B, Al–Ti–C, and Al–Ti–B–C, are utilized in CP-Al with a purity of 99.7%. While these master alloys reduce the grain size of AA1070 with a purity of 99.7%, the Al–B master alloy does not negatively impact electrical conductivity to the same extent as the other master alloys [45]. This indicates that Al–B master alloys are in a more advantageous position among the master alloys. However, it should be noted that Al–B master alloys may play a role in reducing grain size. Therefore, it is crucial to consider a strategy that will reduce the impacts of their grain refinement role while still utilizing the impurity removal capabilities of Al–B master alloys to improve the electrical conductivity of CP-Al. The initial step of this endeavour can be selecting the most suitable master alloy type among Al–B master alloys. In Al–B master alloys containing the AlB_2_ phase, such as Al–3B, the grain-refining effect becomes pronounced. On the other hand, master alloys containing the AlB_12_ phase, such as Al–8B, are more effective for the removal of transition metal impurities [56,57]. Therefore, the use of master alloys such as Al–8B, which contain the AlB_12_ phase, can be considered a more effective approach for the removal of transition metal impurities. However, taking into account the grain-refining properties of the master alloys, it will be necessary to adopt a strategy aimed at increasing the grain sizes. An effective method to achieve this would be to implement a grain-coarsening heat treatment process after the casting process to enlarge the grains in the microstructure of aluminium. From this perspective, the initial step is to add an Al–B master alloy, such as Al–8B, during the casting process. This addition is expected to increase the purity of aluminium, thereby enhancing its electrical conductivity. Subsequently, a grain-coarsening heat treatment will be applied to the aluminium. This treatment will facilitate the formation of a coarse-grained microstructure, leading to further improvements in electrical conductivity.

In this study, the focus is placed on increasing the electrical conductivity of commercially pure aluminium by reducing impurities in the microstructure by adding a master alloy containing boron and then further enhancing the electrical conductivity by increasing the grain size by applying a heat treatment. The objective is to significantly improve the electrical conductivity of commercially pure aluminium through the synergistic effects of boron addition and grain-coarsening heat treatment. Achieving this improvement can greatly enhance the efficiency of induction motors utilizing these materials, thereby leading to increased performance in various types of electric motors used in industrial applications.

## 2. Materials and Methods

### 2.1. Materials

A commercial-grade aluminium with a purity of 99.7% (Eti Alüminyum, Konya, Turkey) was used in the production of squirrel cages of rotors for induction motors in this study. Its chemical composition is presented in Table 1.

To enhance the performance of electric motors, squirrel cage rotors must possess high electrical conductivity; therefore, materials with superior conductivity, such as copper and aluminium, are utilized [58]. When a squirrel cage rotor of an electric motor is fabricated from aluminium, the manufacturing of a copper rotor requires higher processing temperatures due to the elevated melting point of copper. This results in more intense and rapid thermal shock in the electrical steel laminates, significantly reducing rotor efficiency [33,34,35]. Such thermal shock often causes short circuits between the electrical steel laminates and bar conductors, diminishing inter-bar resistance and resulting in inter-bar currents, particularly evident in die-cast copper induction motors [33,36]. The lower melting point of aluminium, in comparison to copper, makes it less likely to encounter these challenges during the manufacturing process of aluminium rotors. In addition to these advantages, commercially pure aluminium with a purity of 99.7% also provides benefits such as low density and cost-effectiveness; consequently, it has become the preferred material for substituting copper in electrical applications [45,59,60]. For all these reasons, this study utilized commercially pure aluminium with a purity of 99.7% for the production of squirrel cage rotors in induction motors.

The purity of aluminium can be enhanced by forming borides with trace elements through the boron present in an added Al–B master alloy, followed by the subsequent removal of these borides, which act as impurities [61]. Al–B master alloys primarily consist of the AlB_2_ and/or AlB_12_ phases. AlB_12_ is less stable than AlB_2_ due to its higher Gibbs free energy; however, this instability allows AlB_12_ to more readily react with impurity metals, forming boride compounds [62]. Consequently, the rate of transition metal removal from molten aluminium is expected to be faster when using AlB_12_-based master alloys compared to those based on AlB_2_ [63]. Thus, Al–B master alloys that include AlB_12_ are more effective and more economic in the removal of impurities compared to those containing AlB_2_ [56,62]. For these reasons, this study utilized the AlB8 master alloy (BDM Bilginoğlu Döküm, Izmir, Turkey), which predominantly contains AlB_12_, to eliminate impurities in aluminium with a purity of 99.7%.

### 2.2. Production Process

**Figure 1:**
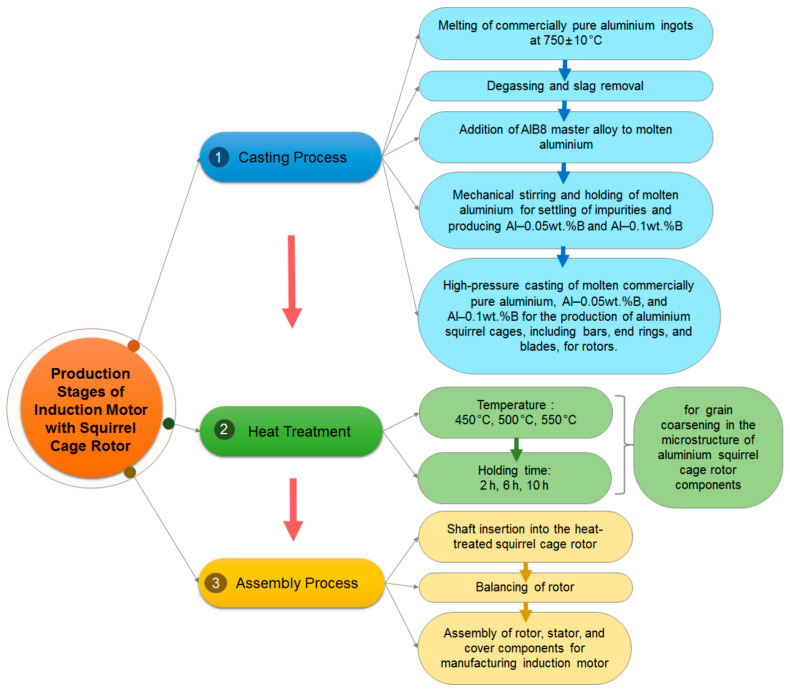
Diagram illustrating the production stages of squirrel cage rotors, heat treatment application, and subsequent manufacturing stages of induction electric motors.

The production stages of the squirrel cage rotors, which encompass the melting and casting of aluminium, followed by the application of heat treatment to the produced rotors, and the subsequent stages of manufacturing the asynchronous (induction) electric motors from these rotors, are shown in Figure 1. The stages of the production processes will be elaborated upon in detail in the subsequent subsections.

#### 2.2.1. Casting

**Figure 2:**
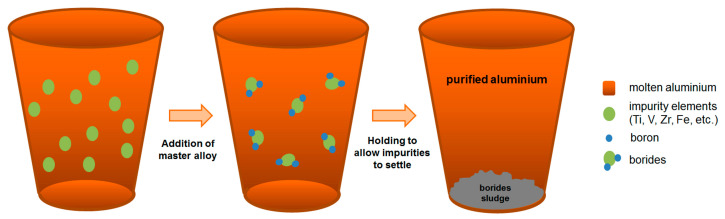
Schematic illustration of impurity removal through boron addition.

In the first stage of the production, commercially pure aluminium (CP-Al) ingots were melted in a crucible induction furnace at 750 ± 10 °C. Slag removal and degassing were performed through the addition of an aluminium silicate-based fluxing powder. The degassing process was carried out for 3 min. Following the degassing process, the slag removal process from the surface of the melt was conducted. To remove impurities present in the melt, the AlB8 master alloy was added in specified amounts, and the melt was mechanically stirred for 5 min. To produce Al–0.05B specimens containing 0.05 wt.% boron, 0.095 kg of the AlB8 master alloy was added into 15 kg of the melted CP-Al ingots. Similarly, for the production of Al–0.1B specimens containing 0.1 wt.% boron, 0.19 kg of the AlB8 master alloy was incorporated into 15 kg of the melted CP-Al ingots. As represented in Figure 2, the addition of the Al–B master alloy to the aluminium melt promotes the formation of boride compounds with trace elements (impurities) present in the melt. Impurities with lower density than aluminium are transferred to the slag, while those with higher density settle at the bottom of the melt in the induction furnace. In order to carry out this purification process, the melt was held at 750 ± 10 °C for 2 h, during which the slag removal process was performed.

**Figure 3:**
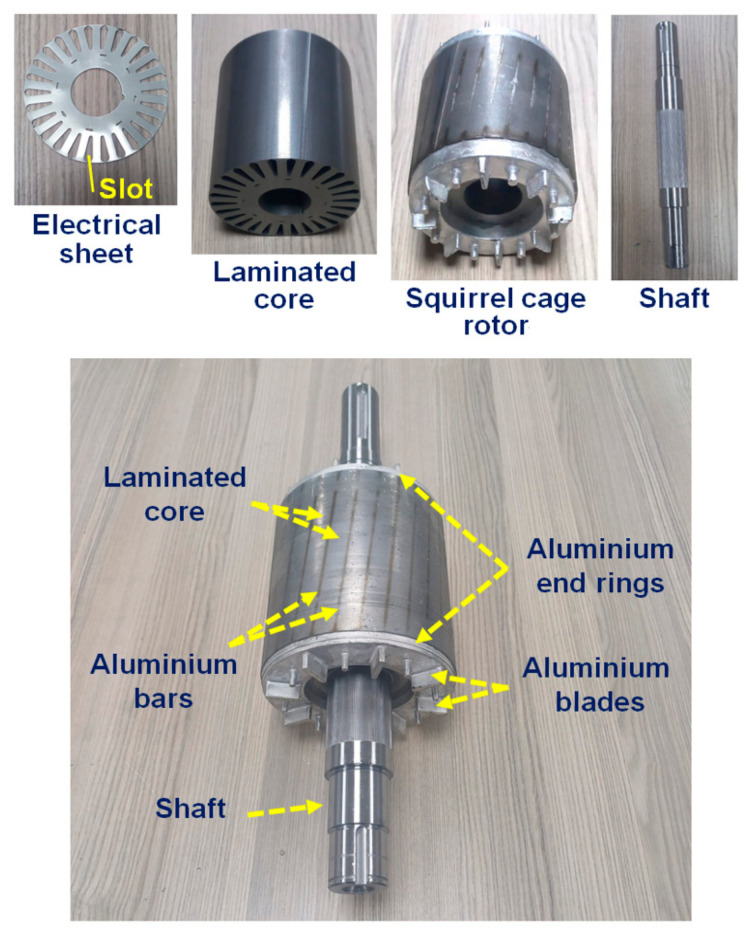
Squirrel cage rotor and its components manufactured from commercially pure aluminium via the high-pressure casting method.

After the completion of the purification process, the casting process was performed using a high-pressure casting machine to produce the squirrel cage structures of the rotors. A squirrel cage rotor, one of the components of induction motors, primarily consists of laminations, rotor/conductor bars, end rings, and a rotor shaft. In the production of the squirrel cage rotors, as illustrated in Figure 3, the steel electrical sheets that form the laminated core of the rotor were first stacked. This lamination served to reduce eddy current losses in the rotor and provided mechanical support for the squirrel cage structure [64,65]. The laminated core featured slots that were aligned according to the design specifications, and it was then placed into the casting mould. The casting mould was configured to facilitate the formation of the squirrel cage structure, including the conductive bars, end rings, and blades. To produce the squirrel cage of the rotor, molten aluminium was poured into the mould containing the laminated core with slots, using a high-pressure aluminium casting process. During this process, the molten aluminium filled the slot cavities to form bars, as well as the sections within the mould cavities to produce end rings and blades. By the end of this process, the squirrel cage of the rotor was successfully manufactured, as illustrated in Figure 3. Subsequently, a shaft was inserted into the rotor, followed by a balancing procedure to ensure its stability. As a result, the rotor was prepared for the production of the induction electric motor.

#### 2.2.2. Heat Treatment

A grain-coarsening heat treatment was performed to the squirrel cage rotors. This process was applied to increase the grain size in the microstructure of purified aluminium, as it was intended to reduce the number of grain boundaries and, consequently, enhance electrical conductivity. In the literature, some studies have applied heat treatment to commercial aluminium with a purity of 99.7%. However, these studies have primarily focused on plastic deformation techniques, such as cold rolling and drawing, followed by annealing heat treatment. In these studies, the heat treatment temperature typically ranged from 200 °C to 400 °C, with holding times varying between 0.5 and 4 h [66,67]. However, this heat treatment type, and consequently, the temperature and duration values, facilitate grain recrystallization. In the present study, the objective was to enhance electrical conductivity by minimizing the total number of grain boundaries. Therefore, a heat treatment method that would significantly promote the increase in grain size was required. To achieve effective grain coarsening, higher heat treatment temperatures should be selected. An upper limit of 550 °C for the heat treatment was established to attain the maximum feasible temperature. This precaution was taken to avoid potential damage to the squirrel cage rotor due to factors such as thermal expansion. In addition to 550 °C, two other temperature values (450 °C and 500 °C) were selected to investigate the effects of elevated temperatures on grain growth and their subsequent influence on electrical conductivity. Another significant parameter affecting grain size is the holding time during heat treatment. For this purpose, preliminary studies were carried out in the production facility. The results showed that as the holding time increased, the grain size increased. However, these preliminary studies revealed that the rate of grain size growth began to slow down after approximately 10 h. Furthermore, extending the holding times would not be deemed inappropriate in terms of energy efficiency and economic considerations. Consequently, the maximum holding time was established at 10 h in the present study. Additionally, to better assess the effect of a 10 h holding time on grain growth, two different holding times (2 h and 6 h) were investigated, with intervals maintained as high as possible. As a result, to study the impact of heat treatment temperature and holding time on grain size within the microstructure, heat treatments were conducted at three different temperatures of 450 °C, 500 °C, and 550 °C for various durations of 2, 6, and 10 h.

#### 2.2.3. Assembly Process

**Figure 4:**
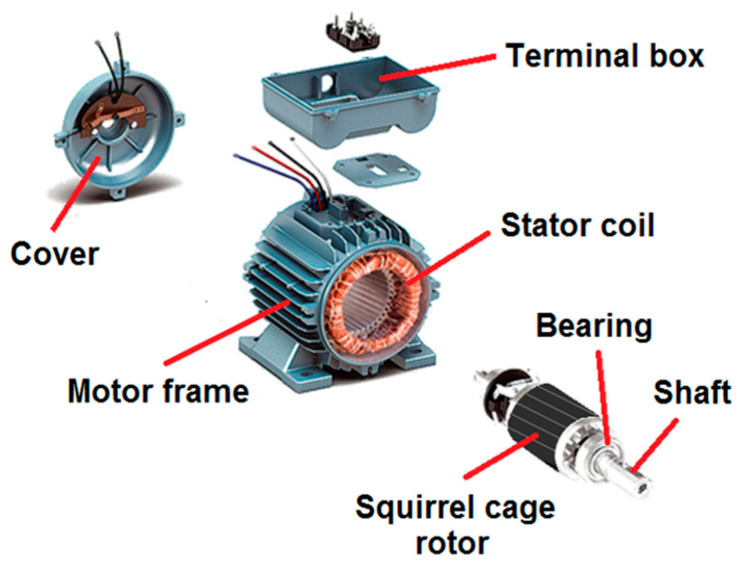
Assembly of induction motor with a squirrel cage rotor.

An induction electric motor consists of a stator, rotor, and frame and a series of assembly stages of these components, as shown in Figure 4.

The construction of the stator in a squirrel cage induction motor involves several critical stages. In the present study, while the production of the previously detailed squirrel cage rotor was in progress, the manufacturing of the stator was conducted concurrently. In the production of the stator, the initial step involved the formation of the stator core (lamination) by stacking steel sheets, aimed at minimizing eddy current losses. This lamination was subsequently assembled into a cylindrical configuration. The next step was the creation of the stator windings, which entailed winding insulated copper wire around the stator slots, specifically designed to accommodate the coils and optimize the distribution of magnetic flux. Typically, the windings were arranged in a three-phase configuration to ensure the generation of smooth and continuous torque. Following the winding process, the coils were secured in position and insulated to avert short circuits. After the coils were placed in the channels and shaped, a varnishing operation was performed to enhance the resistance and protect the wires against abrasion. This process involved curing in an oven for an average duration of 8 h. Subsequently, the stator was inserted into the aluminium frame as the final step in the stator construction. Following this, the front and rear sections of the frame were machined to ensure the proper seating of the motor covers. The frame containing the stator was then transferred to the assembly line. Meanwhile, the bearings were inserted into the shaft of the rotor. Then, the rotor was placed inside the stator. Subsequently, the flange was affixed, followed by the installation of the terminal box and the completion of the electrical connections. Finally, the cover was installed. Once the assembly of all components was completed, a paint application was performed on the motor, which was subsequently cured in an oven to ensure optimal adhesion and finish. Thus, the production process of the induction motor was completed, and the motor was made ready for testing.

### 2.3. Characterization

The chemical compositions of the CP-Al, Al–0.05B, and Al–0.1B specimens were measured using an optical emission spectrometer (Bruker, Karlsruhe, Germany, Q4 TASMAN) to investigate the influence of boron in the AlB8 master alloy on impurity removal and the enhancement in the purity of aluminium. The cast specimens were taken from the middle section of the aluminium melt. The chemical composition analysis was carried out using optical emission spectroscopy due to its high accuracy in measuring a wide range of materials within a microstructure. The analyses were performed at a temperature of 24 ± 1 °C with a humidity level of 50 ± 5%, with five measurements per specimen.

In order to examine the microstructure and grain size of the specimens, an optical microscopy analysis was carried out utilizing an optical microscope (Nikon, Tokyo, Japan, ECLIPSE MA100). For the microstructure analysis, the samples were prepared by grinding with silicon carbide sandpapers (320, 600, 800, 1000, 1500, and 2000 grit) and by polishing with diamond pastes (3 μm, 1 μm, and 0.5 μm); then, they were chemically etched using a solution of 10 mL hydrofluoric acid, 15 mL hydrochloric acid, and 75 mL distilled water for 30 s.

### 2.4. Test Methods

In order to investigate the effects of boron addition and heat treatment on the size of the grains in the microstructure of the specimens, an image processing software tool (ImageJ Version 1.49) was utilized to calculate the grain size. Three microstructure images for each specimen were analysed to determine the grain size, and the average grain size was subsequently calculated.

**Figure 5:**
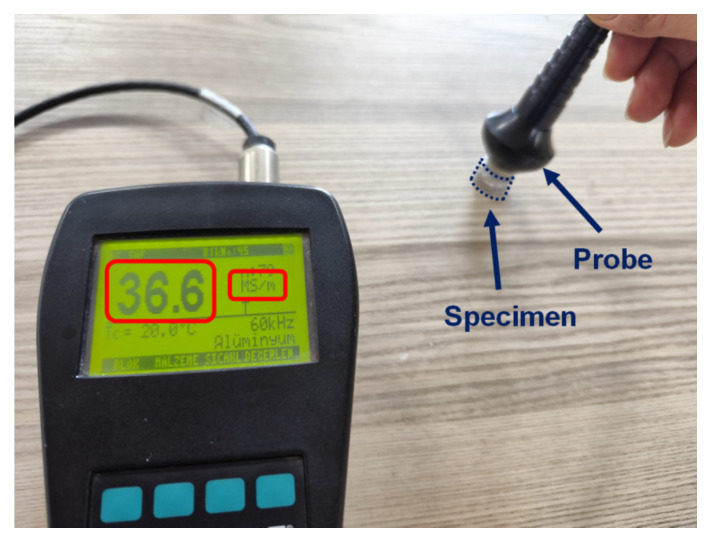
Electrical conductivity measurement for the aluminium specimens (The value and unit within the red rectangle indicates the measured electrical conductivity of a specimen subjected to electrical conductivity testing).

The electrical conductivity of the specimens was measured using an instrument (Fischer, Sindelfingen, Germany, SIGMASCOPE^®^ SMP10) based on the eddy current method according to DIN EN 2004–1 [68] and ASTM E1004–17 [69] (Figure 5). The electrical conductivity of the aluminium specimens was initially measured in MegaSiemens/metre (MS/m) at 20 °C. Three specimens were produced for each combination of experimental parameters, and five measurements were taken for each specimen. In total, fifteen measurements were collected, and the average values for the electrical conductivity were subsequently calculated. The conductivity values were then expressed in terms of the International Annealed Copper Standard (IACS) [70]. As specified in the ASTM E1004 standard, 100% IACS corresponds to an electrical conductivity of 58 MS/m at 20 °C for annealed copper. To convert the average electrical conductivity values of the aluminium specimens from MS/m to %IACS, the conductivity values were divided by 58 MS/m and multiplied by 100. It should be noted that electrical conductivity tests were conducted using the eddy current method. Prior to conducting the electrical conductivity test on the specimens, the specimens were prepared by taking into account the dimension of the calibration block used in the electrical conductivity measurement device. The specimens were machined on a CNC horizontal lathe machine with a tolerance of ±0.001 mm (Hyundai WIA, Changwon, Korea, L200) to ensure that the measuring surface of each specimen was parallel to the opposite surface. Subsequently, the surface of the specimen to be measured with a probe was prepared by eliminating the roughness caused by the lathe tool, resulting in a smooth surface. After this process, the flatness and thickness of the surfaces were re-evaluated using a micrometre with a precision of 0.001 mm. The surfaces of the specimens were finally cleaned, thereby rendering the samples suitable for electrical conductivity measurement.

**Figure 6:**
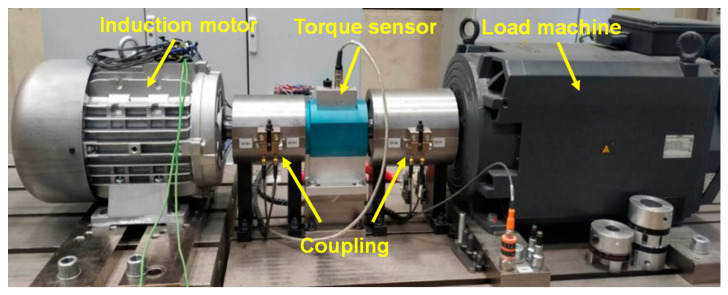
Performance testing of induction motors.

The motor performance test was conducted using motor testing equipment, (KISTLER) in accordance with TS EN 60034–2–1 standard [71], in a laboratory accredited to ISO/IEC 17025 [72], to assess the efficiency of induction motors, as depicted in Figure 6.

According to this method, the tests performed on the motors were as follows: (1) No-load test: The motor is operated without any load connected to its output shaft, and energy losses in this state are measured. (2) Temperature rise test: The motor is run at its nominal power until it reaches a thermal steady state, and the amount of heating is evaluated. (3) Dielectric strength test: A voltage higher than the nominal operating voltage is applied to the motor for a specified period, and the electrical insulation system of the motor is inspected. (4) Over-torque test: A torque higher than the nominal torque of the motor is applied to the output shaft, and the mechanical and thermal stresses on the motor are evaluated. (5) Load curve test: Mechanical loads are applied to the output shaft of the motor using a dynamometer, and the motor performance under these conditions is assessed. These tests verify that the motor operates consistently within specified standards and enable the measurement of motor performance by assessing losses, such as friction, during motor performance tests. In the present study, the motor tests were conducted at a speed of 3000 rpm, over a duration of 3 h. Measurements for motor performance were taken three times for each motor to obtain a more accurate result, and an average value was then calculated.

### 2.5. Experimental Design

Experimental design is a systematic approach that empowers researchers to effectively evaluate the influence of multiple variables or factors on measures of performance or responses. In classical experimental design, the investigation of the effect of a change in one factor on the response is conducted while maintaining all other factors (variables) and their respective values (levels) constant. This methodology is commonly known as the one-factor-at-a-time (OFAT) approach. A significant limitation of the OFAT method is its inability to capture potential interactions among factors and to consider their combined effects. Consequently, this can lead to inaccurate or incomplete conclusions regarding a study. In engineering applications, a diverse array of parameters and their associated values influence the response (output), requiring a substantial number of experiments to gain a thorough understanding of the effects of these factors and to achieve optimization. Design of Experiments (DOE) is a systematic approach for planning and executing experiments aimed at investigating multiple factors and their interactions concurrently. This methodology allows for the simultaneous variation in factors at different levels to evaluate both their individual and combined impacts. Response surface methodology (RSM) is an advanced subset of DOE techniques that facilitates a deeper understanding and optimization of the response. In contrast to classical experimental approaches like OFAT, RSM offers greater efficiency by enabling the simultaneous investigation of multiple factors, thereby minimizing the total number of experiments needed [73]. To investigate the effects of three or more different parameters (factors) and the effects of at least three or more values of these parameters on a property of a material, classical experimental design methods require a substantial number of combinations. For instance, a full-factorial classical experimental design involving three factors (*k*), each at three different levels, results in 27 (3*^k^*) combinations (runs). Furthermore, to ensure the reliability of the experimental results and analyses, it is necessary to perform a specific number of repetitions for each combination. For instance, if three repetitions are conducted for each combination, a total of 81 experiments would need to be carried out. This situation poses time and economic losses, particularly in industrial applications, for research conducted using full-factorial classical experimental design. In this context, fractional factorial design methods, such as central composite design and Box–Behnken design, are recognized as powerful RSMs that significantly minimize the number of experimental runs needed. These methodologies facilitate a thorough investigation of both the main effects and interaction effects of various factors on the response variable. The Box–Behnken design has a smaller number of design points compared to other design methods, such as central composite design, making it more cost-effective to implement with the same number of factors. Additionally, a key advantage of the Box–Behnken design is that it excludes combinations where all factors are simultaneously set to their maximum or minimum levels. This feature helps to prevent conducting experiments under extreme conditions that could lead to undesirable results. For all these reasons [74], the Box–Behnken design was employed in the present study as part of RSM for experimental investigations. This study investigated three factors: the amount of boron, the heat treatment temperature, and the holding time. Table 2 presents these three variables along with their associated low, medium, and high levels, represented by the coded values of −1, 0, and 1, respectively. In order to facilitate regression analysis, the actual (uncoded) values of the variables are coded to improve model fitting and enhance the interpretation of coefficients. To convert the actual values of a variable into their coded equivalents, the following equation (Equation (1)) was used [75]:

(1) $$X_{i}=\frac{x_{i}-x_{i}^{0}}{∆x_{i}},\;\;\;\;\;i=1,\;2,\ldots ,\;k$$

where *X_i_*, *x_i_*, and *x_i_*^0^ represent the coded value, actual (uncoded) value, and the actual value at the centre point of the *i*th independent variable, respectively, and Δ*x_i_* is the step change value of the *i*th independent variable.

**Figure 7:**
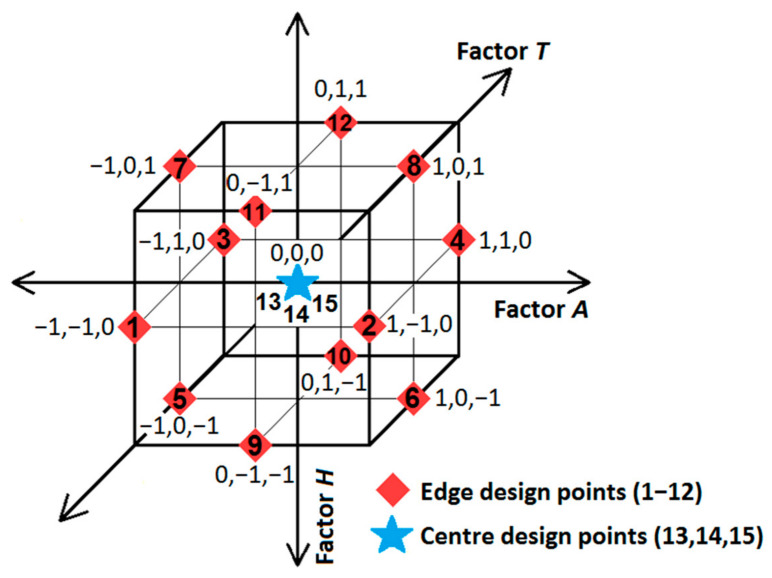
Box–Behnken design cube with the centre point (0,0,0) and twelve factorial points of three factors, each at three levels.

The Box–Behnken design establishes three different levels for each factor (variable) to evaluate the main and interaction effects of the variables on the response. This design method employs a full factorial design with two levels (2*^k^*), systematically incorporating mid-level (0) values between the low (−1) and high (1) levels of the factors to accommodate a greater number of factors. Box–Behnken design consists of the centre point and the middle points of the edges of the experimental space, as shown in Figure 7. The centre point represents the mid-level (0,0,0) combination of each factor, while the twelve middle points of the edges of the experimental space correspond to combination points of three factors, each at three levels.

In this study, the experimental design matrix generated according to the Box–Behnken design method, including both actual and coded values of the factors, is presented in Table 3. The number of experiments (*N*) required in the Box–Behnken design can be calculated as follows:

*N* = 2*k* ∙ (*k* − 1) + *c* (2)

where *k* and *c* are the number of factors and the number of experiment repetitions at the centre point, respectively. Conducting repeated runs at the centre point allows for the estimation of pure error [76]. The number of factors (*k*) is 3, and with 3 repetitions (*c*) conducted at the centre point, this results in a total of 15 experiments (Figure 7 and Table 3), as determined by Equation (2). In Table 3, the first twelve rows of the standard order consist of combinations of particular levels of the three factors, as established by the Box–Behnken design. The last three rows (centre point) of the standard order represent the mid-level (0,0,0) combination for each factor. This mid-level combination was replicated three times to assess the pure error. Experiments were carried out based on the combinations of factor levels in the experimental design matrix created using the Box–Behnken design method (Table 3). However, the experiments were conducted following a randomized run order instead of a standard order, as shown in Table 3. This approach introduces randomness, thereby reducing systematic experimental errors resulting from uncontrolled factors [77,78] (for example, the impact of external factors, such as environmental conditions) and avoiding bias in the response [79], thus enhancing the validity of the results.

The relationship between the response variable and its associated factors is described by the following second-order polynomial equation [75]:

(3) $$Y=\beta_{0}+\sum_{i=1}^{k}\beta_{i}x_{i}+\sum_{i=1}^{k}\beta_{ii}x_{i}^{2}+\;\sum \sum_{i<j=2}^{k}\beta_{ij}x_{i}x_{j}+\varepsilon \;\;i=1,2,\ldots ,k;\;j=2,\ldots ,k$$

where *Y* denotes the response, *β*_0_ is the constant, and *β_i_*, *β_i_*_i_, and *β_ij_* are the linear, quadratic, and interaction coefficients, respectively. *x_i_* and *x_j_* signify the coded levels of factors (*i* and *j* ranging from 1 to *k*), and *k* indicates the number of factors. The term *ε* represents the typical component of random error. In the context of this study, where the model encompasses three factors, the following second-order polynomial equation (Equation (4)), derived from Equation (3), was employed for the prediction of the response:

(4) $$Y=\beta_{0}+\beta_{1}x_{1}+\beta_{2}x_{2}+\beta_{3}x_{3}+\beta_{11}x_{1}^{2}\;+\beta_{22}x_{2}^{2}\;+\beta_{33}x_{3}^{2}+\beta_{12}x_{1}x_{2}+\beta_{13}x_{1}x_{3}+\beta_{23}x_{2}x_{3}+\varepsilon$$

Design Expert Version 13 software was used to analyse the experimental data. An analysis of variance (ANOVA) was conducted to confirm the validity of the second-order polynomial equation representing the response, enabling the evaluation of the significance of each term in the equation and the assessment of the goodness of fit of the model. Furthermore, Design Expert Version 13 software was utilized to generate main effect plots, response surface plots, and contour plots related to the factors.

## 3. Results and Discussion

### 3.1. Spectral Analysis

The addition of boron into molten aluminium promotes the formation of boride compounds by reacting with impurities present in the aluminium. Since the densities of these compounds differ from those of aluminium, they tend to settle at the bottom of the melt. By removing these compounds from the melt, the impurities are effectively eliminated from the microstructure of the aluminium. This phenomenon mitigates the distortion of the lattice structure caused by the presence of impurities, thereby contributing to an enhancement in certain properties of aluminium, such as electrical conductivity [46]. For this purpose, the AlB8 master alloy, which includes AlB_12_, was added to CP-Al to attain boron contents of 0.05% and 0.1% by weight in this study.

The addition of 0.05 wt.% boron into CP-Al was found to positively impact the enhancement in its purity. This was evidenced by a reduction in the amounts of impurity elements in the microstructure of the Al–0.05B specimen, compared to the levels of these elements found in CP-Al (Table 1 and Table 4). The reason for this finding can be explained as follows. The removal of impurities in aluminium depends on the dissolution of boron contained in the master alloy into the melt, which subsequently forms metal–boride (MB) compounds with metallic impurities [80]. This process is influenced by both the type of aluminium–boride (AlB_x_) compound in the master alloy and the characteristics of the impurity elements in the melt. The type of AlB_x_ present in the master alloy, along with the specific metallic impurities that can react, plays a crucial role in the formation of MB compounds [81]. The dissolution of boron contained in a master alloy added to a molten metal depends on the stability of the MB compounds formed between boron and metallic impurities in the melt [80]. If the stability of MB compounds formed in the melt is higher than that of the AlB_x_ compounds present in the master alloy added to the melt, then the boron in the master alloy will continue to dissolve and its reaction with the metallic impurities will continue in the melt. However, if the stability of the MB compounds is lower than that of the AlB_x_ compounds, then the boron in the AlB_x_ compounds will not dissolve and form MB compounds. Gibbs free energy (GFE) values offer a clear approach for assessing the stability of MB compounds. As the GFE decreases, the stability of the MB compounds and their tendency to form will increase. Consequently, AlB_x_ compounds possessing high GFE have the potential to react and form MB compounds with lower GFE [46]. In this study, 0.05 wt.% and 0.1 wt.% boron (B) were added to CP-Al during the casting process. Spectral analyses of the produced Al–0.05B and Al–0.1B specimens revealed a boron content of 0.0103 wt.% and 0.0320 wt.%, respectively (Table 4). These amounts represent the boron retained within the microstructure of specimens, indicating that a substantial amount of the added boron was removed from the aluminium melt during the casting process. The significant removal of the added boron from the melt can be attributed to the formation of MB compounds between boron and metallic impurity elements. This reaction leads to the settling of boron and impurities as MB compounds at the bottom of the melt, thereby facilitating their removal from the microstructure of aluminium [80]. The GFE values of MB compounds, such as TiB_2_, VB_2_, and ZrB_2_, are lower than those of AlB_x_ compounds, indicating that MB compounds are more stable [46] and can be separated from aluminium through gravity settling [82]. Consequently, the addition of the Al–B master alloy into aluminium promotes the formation of MB compounds particularly with Ti, V, and Zr impurities, facilitating their removal from the microstructure through the settling of the aluminium melt [56]. As a result of these phenomena, a significant reduction in the percentages of Ti, V, and Zr impurities in the microstructure of the boron-added aluminium specimens was observed compared to those in CP-Al (Table 1 and Table 4). In the CP-Al, the concentrations of Ti, V, and Zr were measured at 0.0031 wt.%, 0.0042 wt.%, and 0.0016 wt.%, respectively (Table 1). Following the addition of 0.05 wt.%B, these amounts decreased to 0.0012 wt.%, 0.0020 wt.%, and 0.0010 wt.% for Ti, V, and Zr, respectively (Table 4). Consequently, this led to a decrease in the levels of these impurities by 61.3%, 52.4%, and 37.5% for Ti, V, and Zr, respectively. However, the GFE values of Fe borides and Si borides are considerably higher [83,84], complicating the formation of boride compounds of these impurity elements, such as Fe_2_B, and consequently hindering their separation from the aluminium melt. Therefore, adding boron into aluminium cannot be effective for removing impurities such as Fe [83,85]. When comparing the concentrations of Fe and Si present in CP-Al (Table 1), a slight decrease in the percentages of Fe and Si was observed upon the addition of 0.05 wt.%B (Table 4). The reduction in the quantities of Fe and Si could only be achieved by 12.1% and 7.4%, respectively. The fact that the concentrations of Fe and Si in aluminium are higher than those of Ti, V, and Zr may suggest that the removal of these impurities is more critical. However, it is important to note that Ti, V, and Zr have a more significant impact on properties such as the electrical conductivity of aluminium [83,86]. Consequently, despite the lower weight percentages of Ti, V, and Zr compared to Fe and Si, the removal of Ti, V, and Zr is deemed more crucial. In this study, a substantial portion of Ti, V, and Zr impurities was successfully removed from aluminium.

The addition of 0.1 wt.%B into CP-Al was effective in reducing certain impurities such as Ti, V, and Zr. The levels of Ti, V, and Zr were decreased to 0.0018 wt.%, 0.0026 wt.%, and 0.0012 wt.%, respectively, as shown in Table 4. This resulted in reductions in these impurities by 41.9%, 38.1%, and 25%, respectively, compared to their concentrations in CP-Al. Nevertheless, the impact of adding 0.1 wt.%B on the removal of impurities was not as significant as that observed with the addition of 0.05 wt.%B. Furthermore, as seen in Table 4, a significant portion of the added boron remained within the microstructure of Al–0.1B, acting as an impurity and consequently hindering the overall enhancement in aluminium purity. As a result, the addition of 0.1 wt.%B was ineffective in enhancing the purity of CP-Al (99.7%), although it was beneficial in removing certain impurities.

### 3.2. Microstructure

**Figure 8:**
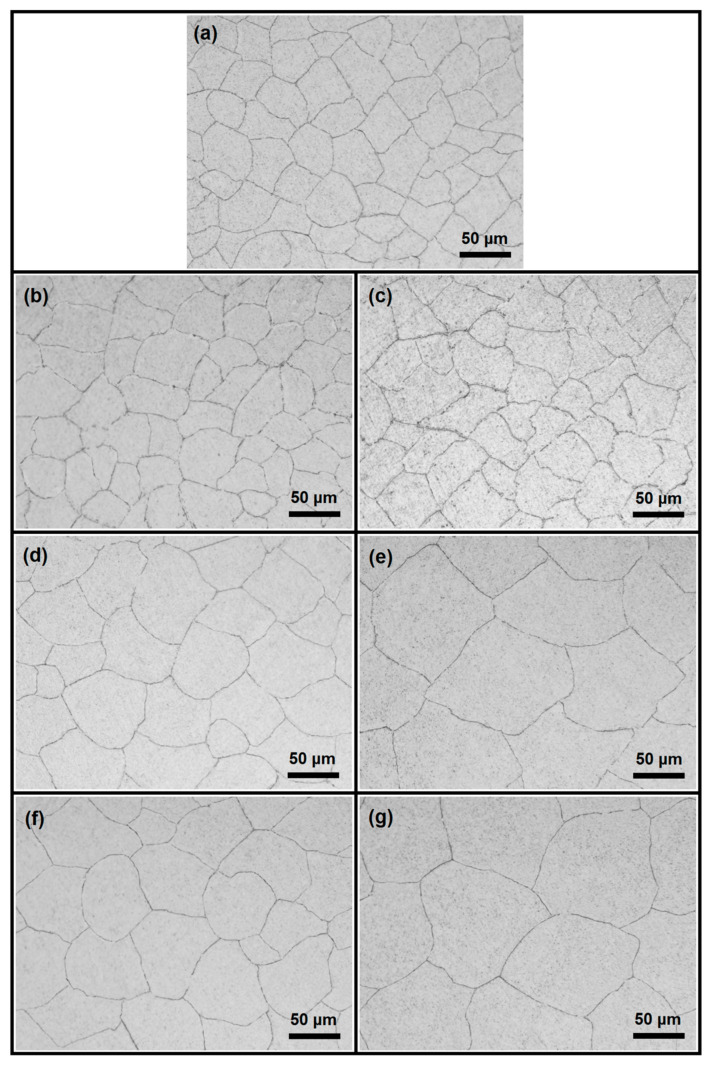
Optical micrographs of the (**a**) non-treated CP-Al, (**b**) non-treated Al–0.05B, (**c**) non-treated Al–0.1B specimens and the heat-treated Al–0.05B specimens at (**d**) 450 °C for 2 h, (**e**) 450 °C for 10 h, (**f**) 550 °C for 2 h, and (**g**) 550 °C for 10 h.

**Figure 9:**
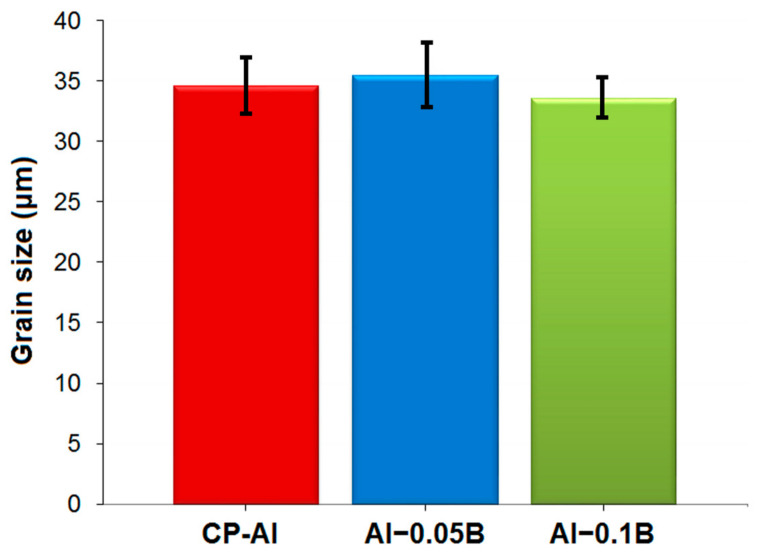
Grain size analysis of the non-heat-treated CP-Al, Al–0.05B, and Al–0.1B specimens.

**Figure 10:**
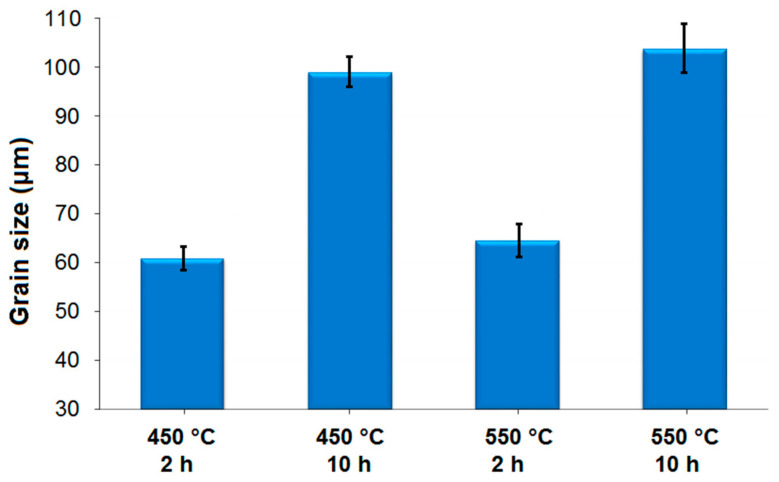
Grain size analysis of the heat-treated Al–0.05B specimens.

The physical properties of metallic materials, such as electrical conductivity, vary depending on their microstructural properties, such as grain size and boundaries. Grain boundaries in the microstructure of a metal induce electron scattering due to their atomic disorder, resulting in decreased electrical conductivity [87,88]. Therefore, grain size can significantly influence the electrical conductivity of metals. To investigate this effect, the present study first analysed the microstructures of the CP-Al, Al–0.05B, and Al–0.1B specimens (Figure 8), followed by the calculation of their corresponding grain sizes (Figure 9 and Figure 10). The mean grain sizes in the microstructures of the CP-Al, Al–0.05B, and Al–0.1B specimens were close to each other (34.6 μm, 35.4 μm, and 33.6 μm, respectively), as seen in Figure 8a–c and Figure 9. However, the grain size in the microstructure of Al−0.1B was smaller than that in the CP-Al and Al–0.05B specimens. This can be attributed to the higher boron content in the Al–0.1B specimen [89] and the lower purity of the Al−0.1B specimen compared to the Al–0.05B specimen (Table 4). The presence of impurities in the Al–0.1B specimen may have enhanced nucleation during the solidification process, leading to a smaller grain size in its microstructure due to the increased nucleation [90].

The electrical conductivity of a pure metal can be enhanced by reducing the number of grain boundaries (or by increasing the size of the grains) in its microstructure. Heat treatment can be an effective method for achieving this. Therefore, in this study, a grain-coarsening heat treatment was applied to the aluminium specimens. Temperature and time in a heat treatment are the most important factors in grain coarsening, as proven in Equations (5) and (6) below [91]:

(5) $$D-D_{0}=Kt^{\frac{1}{n}}$$

where *D* is the grain size to be obtained, *D*_0_ is the initial grain size, *K* is the temperature-dependent constant, *t* is the heat treatment duration, and *n* is the time exponent.

(6) $$K=K_{0}\exp\;\left(-\frac{Q}{RT}\right)$$

where *K*_0_ and *R* are constants, *Q* is the grain growth activation energy, and *T* is the heat treatment temperature. To investigate the impact of the heat treatment on grain size and its subsequent effect on electrical conductivity, the microstructures of the aluminium specimens were analysed. Additionally, a quantitative evaluation of the grain size in the microstructure of the specimens was conducted. Figure 8d–g and Figure 10 present the microstructures and grain sizes of the heat-treated Al–0.05B specimens, respectively. A comparison of the microstructure and grain size between the non-heat-treated Al–0.05B specimen and the heat-treated Al–0.05B specimens revealed that the grain sizes of the heat-treated specimens were significantly coarser (2–3 times larger) than those of the non-heat-treated specimen. This phenomenon can be ascribed to grain growth in metals at elevated temperatures, as heat treatment provides the threshold energy for grain boundaries to migrate, reducing the internal energy and making the microstructure more stable, which leads to an increase in grain size [92].

To comprehensively assess the influence of the heat treatment, the varying impacts of different temperatures and durations on the grain size of the aluminium specimens were also examined. According to the Box–Behnken experimental design (Table 3), the heat treatment was conducted on the Al–0.05B specimens at temperatures of 450 °C and 550 °C for 2 h and 10 h. Compared to the mean grain size of 35.4 μm observed in the non-heat-treated Al–0.05B specimen (Figure 9), the average grain sizes of the heat-treated Al–0.05B specimens were 60.8 μm and 64.5 μm after treatment at 450 °C and 550 °C for 2 h, respectively (Figure 10). This can be attributed to the fact that an increase in temperature enhances atomic mobility, promoting diffusion easily. Consequently, atoms located at grain boundaries migrate from high-energy regions into adjacent grains with lower energy levels, leading to grain merging (grain coarsening) [93]. It should be noted that although the heat treatment of the Al−0.05B specimen resulted in an increase in grain size, the effect of temperature increment during the 2 h treatment was minimal. However, extending the heat treatment duration to 10 h at the temperature of 450 °C led to a grain size of 98.8 μm, indicating a more substantial increase (Figure 10). Similarly, extending the duration from 2 h to 10 h at 550 °C resulted in an increase in grain size from 64.5 μm to 103.6 μm. These findings demonstrated that the duration of the heat treatment had a more pronounced effect on increasing grain size compared to the temperature. The reason for this result can be attributed to the fact that elevated temperatures increase the diffusion rate by raising the atomic kinetic energy.

All results indicated that both the temperature and duration of the heat treatment significantly influenced grain coarsening, which in turn has a beneficial impact on the improvement in the electrical conductivities of commercially pure aluminium, as will be discussed in the subsequent relevant sections.

### 3.3. Electrical Conductivity

Electrical conductivity in metals arises from the movement of electrons. However, lattice vibrations, which depend on temperature, and the interactions between electrons and impurities and defects in the microstructure lead to electron scattering [59,94]. These two factors result in an increase in electrical resistivity, defined as the inverse of conductivity, in metals. According to Matthiessen’s rule (Equation (7)), the electrical resistivity (*ρ*) of metals, due to temperature and microstructure, can be represented as follows [66,95]:

*ρ* = *ρ_T_* + *ρ_R_* (7)

where *ρ_T_* signifies the resistivity caused by the scattering of electrons due to the lattice vibrations, while *ρ_R_* accounts for the resistivity resulting from the scattering by impurities and defects. *ρ_R_* can be expressed as the following equation (Equation (8)) [96]:

*ρ_R_* = *ρ_gb_* + *ρ_d_* + *ρ_ss_* + *ρ_p_* (8)

where *ρ_gb_* represents the resistivity caused by grain boundaries, *ρ_d_* reflects the electron scattering resulting from dislocations, *ρ_ss_* denotes the resistivity attributed to solute or impurity atoms in solid solution, and *ρ_p_* corresponds to the resistivity arising from precipitates of second phases. When a pure metal is alloyed, strain or precipitation hardening leads to a significant reduction in electrical conductivity. Such a reduction is primarily attributed to enhanced electron scattering caused by the increased concentration of solute atoms (impurities) in the matrix, secondary precipitates, and the density of dislocations [97,98]. This phenomenon is more pronounced in alloys that have experienced precipitation (age)-hardening heat treatment and cold plastic deformation processes such as rolling. Therefore, *ρ_ss_*, *ρ_p_*, and *ρ_d_* can significantly influence the electrical resistivity of aluminium alloys that have undergone precipitation heat treatment and plastic deformation [99,100,101,102]. However, in the present study, aluminium with a purity of 99.7% was used, and the casting method was employed as the production process. Thus, the impacts of *ρ_ss_*, *ρ_p_*, and *ρ_d_* on the electrical resistivity of the aluminium specimens can be considered minimal or negligible. The concentrations of impurities were reduced by adding boron compared to those in CP-Al (compare Table 1 to Table 4). Consequently, the addition of boron likely decreased *ρ_ss_* due to its impurity removal effects, leading to an enhancement in electrical conductivity. It should be noted that this approach is applicable to the Al–0.05B specimen; however, the impurity removal influence of boron addition was ineffective in the Al–0.1B specimen due to the relatively higher boron concentration. In contrast to *ρ_ss_*, *ρ_p_*, and *ρ_d_*, it is possible that *ρ_gb_* influenced the resistivity of aluminium as a result of the grain-coarsening heat treatment applied after the casting process. As illustrated in Figure 10, the grain size of the aluminium specimens varied according to the parameters of the heat treatment. With increasing the heat treatment temperature and holding time, the grain size increased. This may have contributed to the enhanced electrical conductivity of aluminium by reducing the number of grain boundaries that cause electron scattering. As a result, the combined effect of the heat treatment and boron addition may have significantly improved the electrical conductivity of aluminium by reducing the *ρ_ss_* and *ρ_gb_* resistivity.

The electrical conductivity of the non-heat-treated CP-Al specimen was 60.62% IACS, which increased to 60.96% IACS with the addition of 0.05 wt.%B to CP-Al aluminium (non-heat-treated Al–0.05B specimen) (Table 5). This improvement can be attributed to the removal of impurities from aluminium through the addition of boron (compare Table 1 to Table 4) [103]. The influence of certain impurities, such as Ti and V, on the electrical conductivity of aluminium, particularly when dissolved in solid solution rather than existing as a precipitate, is notably greater than that of other impurities such as Fe and Si [80,86,104]. Therefore, although the amounts of Ti, V, and Zr are relatively lower in aluminium, their removal is considered more critical for enhancing the electrical conductivity of aluminium. In this study, the addition of 0.05 wt.%B to CP-Al resulted in a high percentage reduction in Ti, V, and Zr impurities (compare Table 1 to Table 4), which contributed to an increase in the electrical conductivity of CP-Al.

The addition of 0.1 wt.%B resulted in a reduction in the concentration of impurities in CP-Al. However, this reduction in the amount of impurities was not as pronounced as that observed with the addition of 0.05 wt.%B (Table 4). It is important to note that Al–B master alloys can serve as effective grain refiners for pure aluminium [50], resulting in a smaller grain size. The reduction in grain size may lead to a negative impact on the electrical conductivity of the material [45,48,105]. In the present study, the grain size of the Al–0.1B specimen was smaller than that of the Al–0.05B specimen, as seen in Figure 8. As a result of these findings, the addition of 0.1 wt.%B was not only ineffective in the improvement in the purity of CP-Al, but it also led to a reduction in grain size. Consequently, the electrical conductivity of the Al–0.1B specimen was lower than that of the Al–0.05B specimen (Table 5).

The electrical conductivity of the CP-Al, Al–0.05B, and Al–0.1B specimens, resulting from the heat treatment applied to the specimens with or without boron at varying temperatures and durations, is presented in Table 6. All results indicated that the application of the heat treatment led to an increase in the electrical conductivity of the CP-Al, Al–0.05B, and Al–0.1B specimens. The electrical conductivity of the CP-Al specimen without boron addition and heat treatment was measured at 60.62% IACS. After applying the heat treatment at 550 °C for 6 h, the electrical conductivity increased to 62.72% IACS. In the Al–0.05B specimen, the heat treatment at 550 °C for 10 h resulted in an increase in electrical conductivity from 60.96% IACS to 63.1% IACS. For the Al–0.1B specimen, the electrical conductivity was enhanced from 60.72% IACS to 62.51% IACS when subjected to the heat treatment at 500 °C for 10 h. These findings also showed that the electrical conductivity of CP-Al increased from 60.62% IACS to 63.1% IACS due to the synergistic effect of the heat treatment and boron addition.

To more comprehensively understand the effects of boron addition, heat treatment, and their interactions, as well as varying values of boron amounts, heat temperatures, and holding times on the electrical conductivity of CP-Al, regression analysis and analysis of variance (ANOVA) were conducted. These analyses were also performed to assess the significance of factors, such as boron addition, heat treatment temperature, and holding time, as well as their interactions, on the response (electrical conductivity), and to verify the adequacy of the model. The model showed a high coefficient of determination (*R*^2^ = 0.9859), revealing that 98.59% of the variability in the response (electrical conductivity) was explained by the factors (boron addition, heat treatment temperature, and holding time) and their interactions in the regression model. The accuracy of the model in predicting the response improves as the *R*^2^ value approaches 1. The adjusted-*R*^2^, which offers an accurate model that fits the existing data, was found to be 0.9605, while the predicted-*R*^2^, indicating the potential accuracy of the model for subsequent data, was 0.8831. The model F-value was 38.83, indicating that the model is significant. Additionally, the F-value for lack of fit was 0.5217, with a *p*-value of 0.7091, suggesting that it is insignificant. This implies that the model fits the data well and is capable of making reliable predictions of the response.

It is important to emphasize that a term is considered significant in the response if its *p*-value is less than 0.05, which corresponds to a 95% confidence level. As seen in Table 7, the *p*-values for the linear terms boron addition (*A*), heat treatment temperature (*T*), and holding time (*H*) were 0.0351, <0.0001, and <0.0001, respectively, while the *p*-values for the quadratic terms boron addition × boron addition (*A*^2^), heat treatment temperature × heat treatment temperature (*T*^2^), and holding time × holding time (*H*^2^) were 0.0266, 0.0022, and 0.0317, respectively. The *p*-values for the interactions of boron addition × heat treatment temperature (*A* × *T*) and heat treatment temperature × holding time (*T* × *H*) were 0.0484 and 0.0233, respectively. All these results reveal that these terms were significant in the response (electrical conductivity). The *p*-value for the interaction between boron addition and holding time (*A* × *H*) was 0.9106, indicating that this interaction was not statistically significant. This suggests that the effect of boron addition on electrical conductivity was independent of holding time, meaning that variations in the holding time did not influence the impact of boron addition on electrical conductivity. The sum of squares value is another expression that indicates the effect of a factor on the response. Factors with higher sum of squares values exert a greater influence on the response. According to the sum of squares, F-values, and *p*-values presented in Table 7, the effects of the terms were as follows: linear > square > interaction. Among the linear terms, the order of effect was *H* > *T* > *A*; for the square terms, it was *T*^2^ > *A*^2^ > *H*^2^; and for the interactions, it was *T* × *H* > *A* × *T* > *A* × *H*. It should be noted that there was no significant interaction between boron addition and holding time (*A* × *H*) on the response, as previously mentioned.

**Figure 11:**
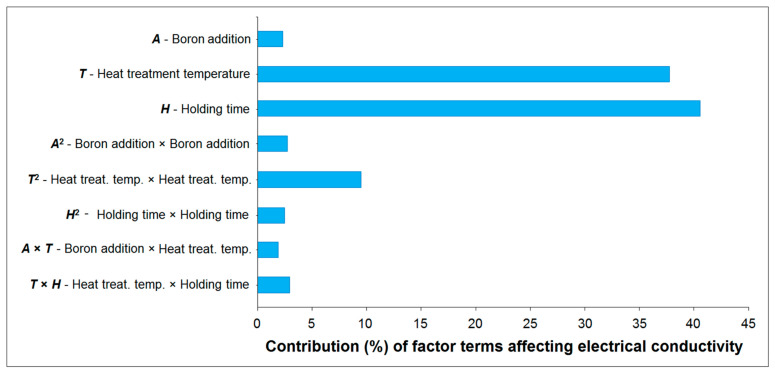
Contributions of linear, quadratic, and interaction terms affecting electrical conductivity.

The predictive capability of the model was enhanced by excluding the insignificant *A* × *H* term. Subsequently, the contributions and coefficients of the linear, quadratic, and interaction terms that significantly influenced the response (electrical conductivity) of the factors were analysed, as presented in Table 8 and Figure 11. The contribution of a term to the response was calculated by dividing its sum of squares by the total sum of squares. Heat treatment temperature was the most influential factor for electrical conductivity among the factors, followed by holding time and boron addition. When the *A* × *H* term that did not significantly affect the electrical conductivity was removed from the analysis, the prediction capability (predicted-*R*^2^) of the model increased from 88.31% to 91.99%.

**Figure 12:**
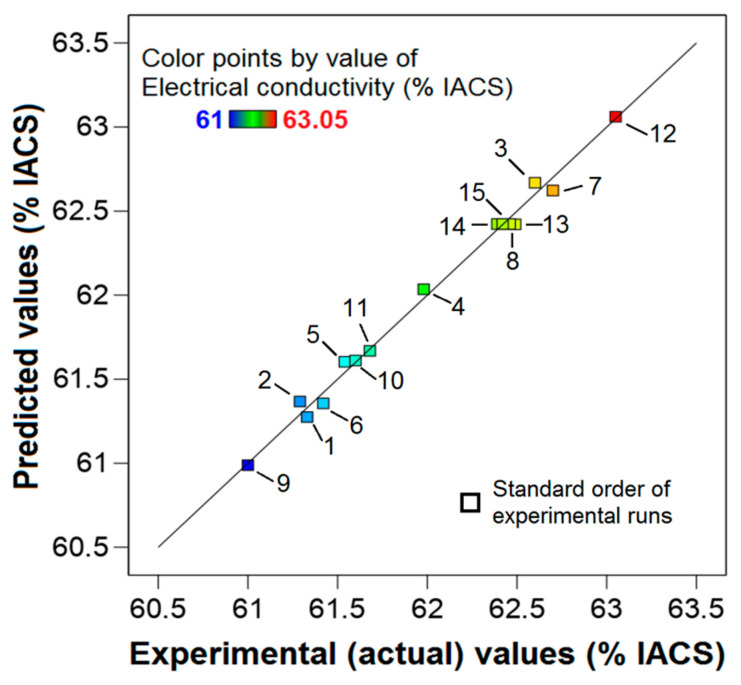
Correlation graph between the predicted and experimental values of electrical conductivity (Each number indicates the standard order of experimental runs).

The regression equation for the refined model, which includes only the coefficients of significant terms in coded values, excluding the insignificant interaction term *A* × *H*, is presented as follows:

*EC* (%) = 62.42 − 0.1288 × *A* + 0.52 × *T* + 0.5388 × *H* − 0.2054 × *A*^2^ − 0.3829 × *T*^2^ − 0.1954 × *H*^2^ − 0.165 × *A* × *T* + 0.205 × *T* × *H* (9)

where the presence of a positive sign in a term coefficient indicates a synergistic effect, while a negative sign implies an antagonistic effect. As seen in Equation (9), among the linear terms, heat treatment temperature (*T*) and holding time (*H*) were found to be positive, and the interaction of these two terms (*T* × *H*) was also positive, indicating a positive correlation with the response (electrical conductivity). However, boron addition (*A*) and all the quadratic terms (*A*^2^, *T*^2^, and *H*^2^) were negative. It should be noted that while the addition of boron had a negative coefficient in Equation (9), its increase up to a certain level enhanced electrical conductivity. However, at a higher level of boron addition, a negative effect on electrical conductivity was observed (Table 6), which aligns with the negative coefficient in Equation (9). Figure 12 illustrates a scatter plot that contrasts the predicted and experimental electrical conductivity values. The actual data points aligned closely with the regression line, indicating a good fit for the model.

**Figure 13:**
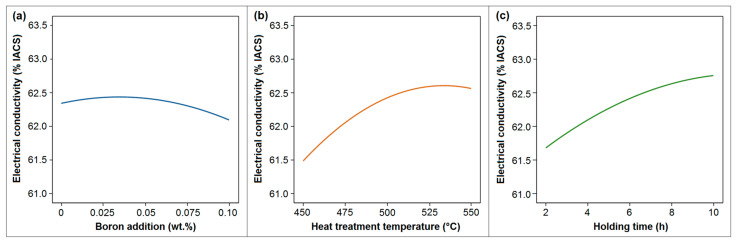
Main effect plots of (**a**) boron addition, (**b**) heat treatment temperature, and (**c**) holding time on electrical conductivity.

The main effect plot is a graphical representation for observing the main effect of factors on the response. Figure 13 depicts the individual main effects of boron addition, heat treatment temperature, and holding time on electrical conductivity. Heat treatment temperature and holding time were significant factors influencing electrical conductivity (Figure 13b,c), while the addition of boron had a minor effect on this property (Figure 13a). An increase in temperature led to a continuous enhancement in conductivity; however, the rate of increase in temperature gradually diminished (Figure 13b). A similar influence was observed for holding time (Figure 13c). The positive impact of elevated temperature and extended holding time on the enhancement in electrical conductivity can be attributed to the increase in grain size within the microstructure resulting from the higher values of these factors (Figure 8 and Figure 10). The addition of boron resulted in a slight increase in electrical conductivity, attributed to its effectiveness in removing impurities from the microstructure and enhancing the purity of the material. However, as boron addition increased, residual boron and its compounds remained within the microstructure, leading to a decrease in purity and a corresponding reduction in electrical conductivity.

**Figure 14:**
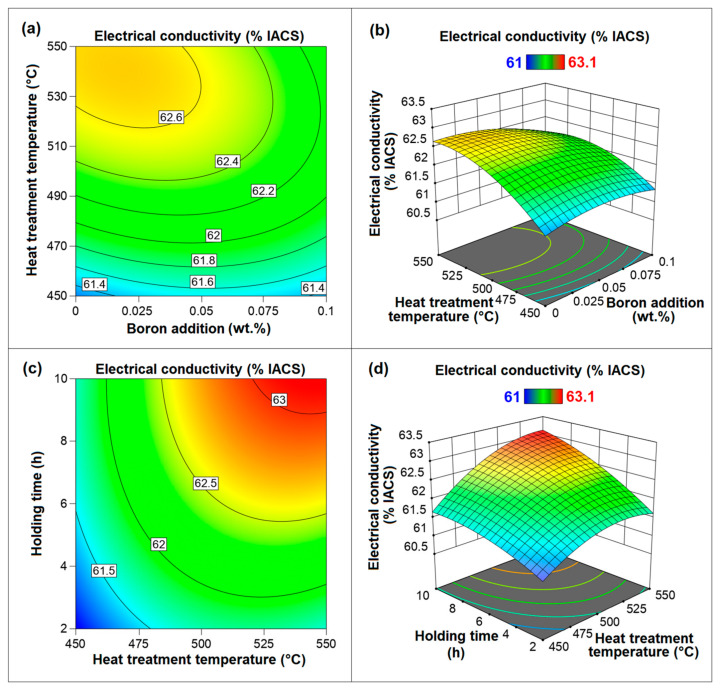
(**a**) Two-dimensional contour plot and (**b**) 3D response surface plot showing the effect of the interaction (*A* × *T*) between boron addition and heat treatment temperature and (**c**) 2D contour plot and (**d**) 3D response surface plot showing the effect of the interaction (*T* × *H*) between heat treatment temperature and holding time on the electrical conductivity of commercially pure aluminium.

Three-dimensional response surface curves and 2D contour charts illustrate regression equations, providing a clearer perspective on the interaction between factors and their impact on the response. It is important to note that while a response surface curve and a contour plot show the effect of any two factors on the response, the values of the other factors are held at their coded 0 (middle) level (value) or the actual (uncoded) middle value. The effects of boron addition, heat treatment temperature, and holding time and their interactions on electrical conductivity are illustrated in Figure 14. The interaction between boron addition and heat treatment temperature (*A* × *T*) and the interaction between heat treatment temperature and holding time (*T* × *H*) were found to be significant, whereas the interaction between boron addition and holding time (*A* × *H*) had insignificant effect on the response according to the ANOVA results in Table 7. Therefore, 3D response surface curves and 2D contour charts of interactions *A* × *T* and *T* × *H* were investigated (Figure 14). The highest values of electrical conductivity were achieved with relatively low boron addition and elevated heat treatment temperatures (Figure 14a,b). An increase in heat treatment temperature, at any level of boron addition, had a positive effect on electrical conductivity. On the other hand, at high levels of heat treatment temperature, increasing boron addition negatively impacted electrical conductivity, whereas at low levels of heat treatment temperature, there was no significant effect from increased boron addition. According to the ANOVA results (Table 7 and Table 8), another interaction that influenced electrical conductivity was the interaction between heat treatment temperature and holding time (Figure 14c,d). At all heat treatment temperature levels, electrical conductivity increased with longer holding times. An increase in heat treatment temperature at any holding time level exhibited a similar effect. At higher values of both temperature and holding time, their combined contributions to enhancing electrical conductivity were more pronounced.

### 3.4. Motor Efficiency

**Figure 15:**
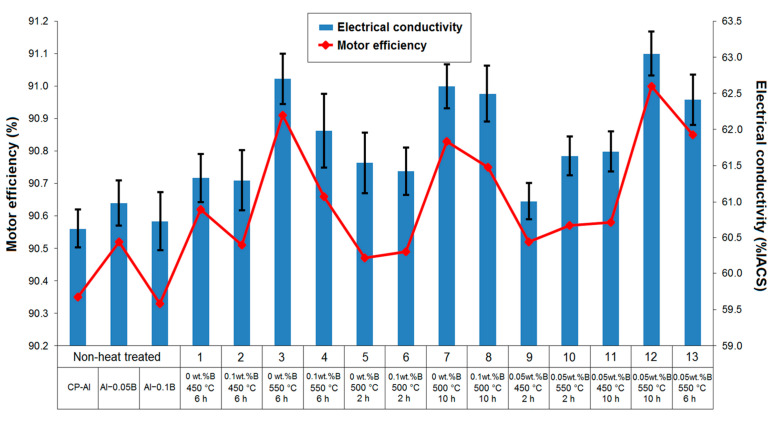
Relation between the motor efficiency and electrical conductivity of non-treated and heat-treated CP-Al, Al–0.05B, and Al–0.1B.

The efficiency of induction electric motors is significantly influenced by their components, such as the rotor, and the electrical conductivity of the materials, such as aluminium, used in these components [106]. An enhancement in the electrical conductivity of CP-Al used for rotor production aims to increase the efficiency of induction electrical motors, as aforementioned in this study. To this end, the relationship between electrical conductivity and motor efficiency was investigated. As illustrated in Figure 15, there was a strong correlation between electrical conductivity and motor efficiency, with motor efficiency exhibiting an increasing trend similar to that of electrical conductivity. The efficiency of the motors produced from the heat-treated CP-Al rotors was higher than those manufactured from the non–heat-treated CP-Al. The efficiency of the motor produced from the rotor made of CP-Al was measured at 90.35%. However, by adding 0.05 wt.%B into CP-Al, the efficiency of the motor constructed from the rotor made of this modified material was increased to 90.52%. The efficiency of the motor produced with a rotor made from CP-Al was improved from 90.35% to 91.21% through the application of the heat treatment to the rotor (specimen no 3). When comparing the efficiency of the motor produced from the CP-Al rotor, both the addition of 0.05 wt.% boron and the application of the heat treatment to the rotor (specimen no 12) resulted in a synergistic effect, raising the motor efficiency from 90.35% to 91.53%. As a result, these findings indicate that the addition of boron to CP-Al and the application of the heat treatment to the rotors indirectly but significantly influenced the improvement in the efficiency of the induction electric motors.

This study has introduced a simple and effective hybrid approach that enhances the electrical conductivity of commercially pure aluminium through the addition of boron (AlB8 master alloy) and the application of a grain-coarsening heat treatment. This innovative hybrid approach facilitates a reduction in the impurities in the microstructure of commercially pure aluminium due to the addition of boron, while also decreasing grain boundary density as a result of the heat treatment. As a result, the electrical conductivity of commercially pure aluminium increased from 60.62% IACS to 63.1% IACS. This enhancement renders the material suitable for applications that require this level of electrical conductivity, where mechanical loading is not a primary consideration. The modifications achievable in the microstructure of this material suggest potential enhancements not only in electrical conductivity but also in thermal conductivity and corrosion resistance. The components manufactured from the materials produced through this method will improve the energy efficiency, performance, and durability of the machines constructed from these components. This advancement will yield substantial economic and environmental benefits, thereby contributing to the overall enhancement of industrial processes and product quality. It is also important to emphasize that commercially pure aluminium, with increased electrical conductivity achieved through the method presented in this study, could serve as a cost-effective alternative to high-purity aluminium, which is highly conductive but expensive and challenging to supply globally, particularly for industrial-scale production. Consequently, it has significant promise for a wide range of applications, including electrical motors in electrical applications, vehicle components in the automotive industry, and heat exchangers in the energy sector for large-scale industrial production.

## 4. Conclusions

This study investigated the effects of adding an AlB8 master alloy to commercially pure aluminium (CP-Al) and applying a grain-coarsening heat treatment on the microstructural properties and electrical conductivity. Furthermore, the efficiency of motors constructed from the rotors produced from CP-Al was determined. Based on the findings, the following conclusions can be drawn:

- The addition of 0.05 wt.% boron (B) to CP-Al was effective in reducing impurities within the microstructure of CP-Al. The incorporation of 0.1 wt.%B led to a decrease in impurity concentrations within CP-Al. However, this reduction was less significant compared to the effect observed with the addition of 0.05 wt.%B. Additionally, some of the boron from the AlB8 master alloy persisted within the CP-Al, which caused the addition of 0.1 wt.%B to be ineffective in increasing the purity of CP-Al. As a result, the addition of 0.05 wt.%B enhanced the electrical conductivity of CP-Al; however, the incorporation of 0.1 wt.%B did not yield a similar improvement in the electrical conductivity of CP-Al.
- The application of heat treatment had a great impact on the improvement in the electrical conductivity of CP-Al. As the holding temperature rose from 450 °C to 550 °C and the holding time increased from 2 h to 10 h, the electrical conductivity of the rotors consistently improved due to the increase in the size of the grains within the microstructure. However, this positive effect continued at a diminishing rate as the holding temperature and time were further enhanced.
- Compared to the effect of adding AlB8, the heat treatment had a more significant influence on enhancing the electrical conductivity of CP-Al. The electrical conductivity of CP-Al with an initial conductivity of 60.62% IACS was enhanced by the addition of 0.05 wt.%B, which increased the conductivity to only 60.96% IACS. However, the subsequent heat treatment resulted in a synergistic effect between the boron addition and the thermal process, leading to a further increase in conductivity to 63.1% IACS.
- The motor efficiency of a rotor produced from CP-Al was determined to be 90.35%. The addition of 0.05 wt.%B increased the motor efficiency from 90.35% to 90.52%. Applying the heat treatment to the CP-Al rotor enhanced the efficiency to 91.21%. Notably, the combination of adding the AlB8 master alloy and applying the heat treatment resulted in a synergistic effect, further increasing motor efficiency to 91.53%.

As a result of the innovative hybrid approach presented in this study, the electrical conductivity of commercially pure aluminium has significantly increased, making it a potential candidate for industrial applications where high electrical conductivity is essential, such as in electric motors. Consequently, this method can also be utilized to enhance the purity of commercially pure aluminium with lower purity levels than those investigated in this study. However, it is important to note that the inadequate mechanical properties of pure aluminium, despite its high electrical conductivity, can limit its applicability in situations subjected to high mechanical loading. Therefore, future research will concentrate on enhancing the mechanical properties of commercially pure aluminium, which has had its electrical conductivity improved through the hybrid method applied in this study, thereby further diversifying and expanding its potential applications.

## Figures and Tables

**Table 1:** Chemical composition of the commercially pure aluminium (99.7%).

	Elements											
	Fe	Si	Zn	Cu	Ti	Ni	Zr	Cr	V	Mg	Mn	Al
Commercially pure aluminium	0.1865	0.0885	0.0046	0.0030	0.0031	0.0009	0.0016	0.0015	0.0042	0.0003	0.0002	Bal.

**Table 2:** Factors used in the experimental design, their actual values, and their corresponding coded levels.

Factor	Symbol		Level		
	Actual	Coded	−1	0	1
Boron addition (wt.%)	*a*	*A*	0	0.05	0.1
Heat treatment temperature (°C)	*t*	*T*	450	500	550
Holding time (h)	*h*	*H*	2	6	10

**Table 3:** Experimental design matrix using the Box–Behnken design including the actual values and coded levels of factors.

Standard Order	Run Order	Coded Level			Actual (Uncoded) Value		
		*A*	*T*	*H*	*a*	*t*	*h*
1	6	−1	−1	0	0	450	6
2	4	1	−1	0	0.1	450	6
3	3	−1	1	0	0	550	6
4	14	1	1	0	0.1	550	6
5	15	−1	0	−1	0	500	2
6	8	1	0	−1	0.1	500	2
7	11	−1	0	1	0	500	10
8	10	1	0	1	0.1	500	10
9	12	0	−1	−1	0.05	450	2
10	5	0	1	−1	0.05	550	2
11	9	0	−1	1	0.05	450	10
12	7	0	1	1	0.05	550	10
13	2	0	0	0	0.05	500	6
14	1	0	0	0	0.05	500	6
15	13	0	0	0	0.05	500	6

**Table 4:** Chemical composition (wt.%) of Al–0.05B and Al–0.1B.

		Elements (wt.%)											
	Fe	Si	Zn	Cu	Ti	Ni	Zr	Cr	V	Mg	Mn	B	Al
Al–0.05B	0.1638	0.0812	0.0053	0.0021	0.0012	0.0007	0.0010	0.0012	0.0020	0.0002	0.0002	0.0103	Bal.
Al–0.1B	0.1727	0.0831	0.0043	0.0024	0.0018	0.0010	0.0012	0.0016	0.0026	0.0002	0.0002	0.0320	Bal.

**Table 5:** Electrical conductivity of the non-heat-treated CP-Al, Al–0.05B, and Al–0.1B specimens.

	Non-Heat-Treated Specimens		
	CP-Al	Al–0.05B	Al–0.1B
Electrical conductivity (%IACS)	60.62 ± 0.28	60.96 ± 0.35	60.73 ± 0.44

**Table 6:** Experimental electrical conductivity results of the aluminium specimens produced from various combinations of boron addition, heat treatment temperature, and holding time according to the Box–Behnken design.

Standard Order	Boron Addition (wt.%)	Heat Treatment Temperature (°C)	Holding Time (h)	Electrical Conductivity (MS/m)	Electrical Conductivity (% IACS) *EC*
1	0	450	6	35.58	61.34
2	0.1	450	6	35.54	61.28
3	0	550	6	36.38	62.72
4	0.1	550	6	35.96	62.00
5	0	500	2	35.70	61.55
6	0.1	500	2	35.62	61.41
7	0	500	10	36.32	62.62
8	0.1	500	10	36.26	62.51
9	0.05	450	2	35.38	61.00
10	0.05	550	2	35.74	61.62
11	0.05	450	10	35.76	61.66
12	0.05	550	10	36.60	63.10
13	0.05	500	6	36.30	62.58
14	0.05	500	6	36.12	62.28
15	0.05	500	6	36.20	62.41

**Table 7:** Analysis of variance (ANOVA) for electrical conductivity depending on the factors of boron addition, heat treatment temperature, and holding time.

Source	Degree of Freedom (DF)	Sum of Squares	Mean Square	F-Value	*p*-Value
Model	9	5.64	0.6266	38.83	0.0004
*A*—Boron addition	1	0.1326	0.1326	8.22	0.0351
*T*–Heat treatment temperature	1	2.16	2.16	134.04	<0.0001
*H*—Holding time	1	2.32	2.32	143.88	<0.0001
*A* ^2^	1	0.1558	0.1558	9.65	0.0266
*T* ^2^	1	0.5414	0.5414	33.55	0.0022
*H* ^2^	1	0.1410	0.1410	8.74	0.0317
*A* × *T*	1	0.1089	0.1089	6.75	0.0484
*A* × *H*	1	0.0002	0.0002	0.0139	0.9106
*T* × *H*	1	0.1681	0.1681	10.42	0.0233
Residual	5	0.0807	0.0161		
Lack of fit	3	0.0354	0.0118	0.5217	0.7091
Pure error	2	0.0453	0.0226		
Total	14	5.72			

**Table 8:** Coefficients of linear, quadratic, and interaction terms of factors influencing the response.

Term	Coefficient Estimate	DF	Standard Error of Coefficient	*p*-Value
Intercept	62.42	1	0.0670	<0.0001
*A*	−0.1288	1	0.0411	0.0202
*T*	0.5200	1	0.0411	<0.0001
*H*	0.5388	1	0.0411	<0.0001
*A* ^2^	−0.2054	1	0.0604	0.0145
*T* ^2^	−0.3829	1	0.0604	0.0007
*H* ^2^	−0.1954	1	0.0604	0.0178
*A* × *T*	−0.1650	1	0.0581	0.0295
*T* × *H*	0.2050	1	0.0581	0.0124

## Data Availability

The original contributions presented in this study are included in this article; further inquiries can be directed to the corresponding author.
